# Spatial Frequency Domain Imaging System Calibration, Correction and Application for Pear Surface Damage Detection

**DOI:** 10.3390/foods10092151

**Published:** 2021-09-11

**Authors:** Yifeng Luo, Xu Jiang, Xiaping Fu

**Affiliations:** Faculty of Mechanical Engineering and Automation, Zhejiang Sci-Tech University, Hangzhou 310018, China; 15555437863@163.com (Y.L.); jiangxu079@163.com (X.J.)

**Keywords:** spatial frequency domain imaging (SFDI), projector-camera calibration, optical properties, pears, damage detection, linear discriminant analysis (LDA)

## Abstract

Spatial frequency domain imaging (SFDI) is a non-contact wide-field optical imaging technique for optical property detection. This study aimed to establish an SFDI system and investigate the effects of system calibration, error analysis and correction on the measurement of optical properties. Optical parameter characteristic measurements of normal pears with three different damage types were performed using the calibrated system. The obtained absorption coefficient *μ_a_* and the reduced scattering coefficient *μ’_s_* were used for discriminating pears with different surface damage using a linear discriminant analysis model. The results showed that at 527 nm and 675 nm, the pears’ quadruple classification (normal, bruised, scratched and abraded) accuracy using the SFDI technique was 92.5% and 83.8%, respectively, which has an advantage compared with the conventional planar light classification results of 82.5% and 77.5%. The three-way classification (normal, minor damage and serious damage) SFDI technique was as high as 100% and 98.8% at 527 nm and 675 nm, respectively, while the classification accuracy of conventional planar light was 93.8% and 93.8%, respectively. The results of this study indicated that SFDI has the potential to detect different damage types in fruit and that the SFDI technique has a promising future in agricultural product quality inspection in further research.

## 1. Introduction

During the collection and transportation of fruit, collisions are likely to occur, causing surface defects such as bruises, scratches and abrasions, while damaged fruits are prone to decay and affect other normal fruits. To reduce unnecessary losses, it is essential to grade fruits according to the degree of damage suffered. At present, the commonly used rapid and non-destructive detection methods are mainly visible light imaging and hyperspectral imaging techniques based on reflectance measurements [1,2], and Raman techniques based on inelastic scattering [3]. However, these techniques find it difficult to achieve the detection of fine defects and initial damage in fruits [4,5].

In recent studies, it has been suggested that the degree of small bruises in fruits can be studied quantitatively by studying the variation in the obtained absorption coefficient *μ_a_* and the reduced scattering coefficient *μ’_s_* [6]. At present, the commonly used methods for the detection of optical characteristic parameters mainly include the integrating sphere technique [7], the time-resolved (TR) technique [8] and the spatially resolved (SR) technique [9]. The integrating sphere technique is a destructive measurement method, and the sample preparation process is tedious; it cannot achieve rapid non-destructive measurements and it is often used as a reference method. TR techniques have high measurement accuracy, but the system is expensive and requires the probe to be in contact with the sample, which is difficult to apply in damage detection in fruits. SR technique can only perform point measurements, although the equipment is cheaper and can be non-destructive. These methods were described in detail in a recent review by Lu et al. and can be consulted by those interested [10].

Spatial frequency domain imaging (SFDI) is a non-contact wide-field optical imaging technique, which was first proposed by Cuccia et al. [11]. This technique uses structured light and specific optical transmission models to quickly and non-destructively acquire the large-area optical properties of tissue objects. The principle of the technique is based on diffusion theory, introducing a spatially modulated source into the steady-state diffusion equation and realizing spatial resolution measurements. In the past 10 years, SFDI has been mainly used in medical and biomedical fields, such as skin disease detection [12,13], blood oxygen content detection [14,15], pig skin burn level detection [16], etc. In recent years, SFDI has been applied to agricultural product quality detection, such as apple bruise detection [17] and pear bruise detection [18,19].

In an SFDI system, the basic components include a projection device, a collection device, optical components (polarizers, filters, neutral density filters, etc.) and so on. The projection device can be a commercial projector or illumination components with light emitting diodes (LEDs) [20,21]. The advantage of using illumination components is that a collimated light source can be used and the fringe frequency accuracy is higher, while the disadvantage is that the fringe frequency set by the optical grating is difficult to change. The advantage of using a commercial projector is that changing the fringe frequency is easier, while the disadvantage is that there is keystone distortion. After eliminating the projection errors, the commercial projector is more suitable for SFDI research.

In optical imaging systems using a commercial projector as a light source, including SFDI systems, the main external error sources include: asynchronous acquisition and projection frame rates, keystone distortion of the projection [22], non-linearity of the projection intensity and image acquisition [23,24], and lens distortion [25], etc. The mismatch between the refresh frame rate of the projector and the capture frame rate of the camera will introduce additional harmonics in the captured image. Commercial projectors are usually optimized for human vision, which results in the projected pattern being no longer sinusoidal and not being able to be correctly demodulated, and the non-linearity of the projection needs to be corrected. The keystone of the projection is mainly caused by the angle between the projector and the projection plane. Because the beam of the projector is non-collimated, the distance between the projection plane and the projector will affect the frequency of the sinusoidal patterns projected on the sample. Therefore, calibration of the SFDI system is a practical need and is important to reduce the system error and achieve accurate measurement of the target. The main objective of this study was to calibrate the system for SFDI and to use the calibrated SFDI system to obtain the optical characteristics of pears for surface damage discrimination. The specific objectives of this study were (1) to develop a SFDI system and its control software that can be used for optical property measurements; (2) to perform projector–camera calibration of the SFDI system, and perform keystone correction and frequency calibrations based on the calibration results; (3) to use liquid phantoms to calibrate the measurement errors of the system and calculate the *μ_a_* and *μ’_s_* of ‘crown’ pears; (4) to discriminate the normal pears and pears with different damage types based on the obtained *μ_a_* and *μ’_s_*.

## 2. Materials and Methods

### 2.1. SFDI System Construction

#### 2.1.1. Hardware

**Figure 1:**
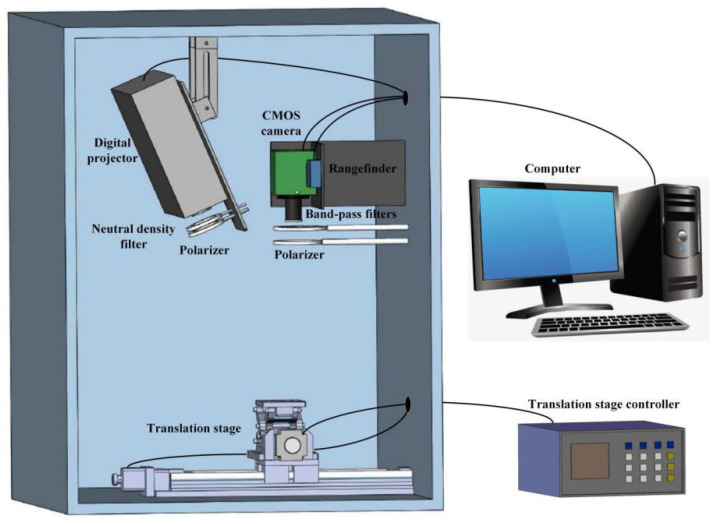
Schematic diagram of the developed SFDI system.

A schematic diagram of the developed SFDI system is illustrated in Figure 1. It mainly includes a camera, a filter wheel, two polarizers, a rangefinder, a projector, a sample stage and its controller, a computer and other fixtures. The system was built in a black box with dimensions of 1000 × 1000 × 1000 mm^3^. It is a wide-field imaging system using a 16-bit high-performance Scientific Complementary Metal-Oxide-Semiconductor (sCMOS) camera (Iris 9TM, Photometrics, Inc., Tucson, AZ, USA). The area array of the sCMOS camera is 2960 × 2960 pixels, and the peak quantum efficiency (QE) is greater than 73%. During the camera acquisition process, the sCMOS camera is cooled down to around 0 °C at an ambient temperature of 30 °C to minimize dark current. The camera was connected to the computer through the manufacturer’s paired peripheral component interconnect express (PCI-Express) capture card, which simultaneously implemented camera control and data transmission, and greatly increased the transmission speed of 16-bit image data. A large-aperture lens (LEM2520CD-H2, Vision Datum Co., Ltd., Hangzhou, China) was mounted on the camera. There was a filter wheel (FW102, Beijing Optical Century Instrument Co., Ltd., Beijing, China) and a polarizer (OCZ203, Beijing Optical Century Instrument Co., Ltd., Beijing, China) in front of the lens. Six band-pass filters (Mega-9 Co., Ltd., Shanghai, China) were installed in the filter wheel. The distance between the camera and the sample stage was about 400 mm, and the field of view was about 150 × 150 mm^2^. There was a rangefinder (CD33, Optex Co., Ltd., Shiga, Japan) on the side of the camera to measure the distance from the sample to the camera in the vertical direction, which affected the frequency calibration.

The light source was a Nippon electronic company (NEC) projector (NEC NP-V302WC, NEC Corporation, Tokyo, Japan), and the color wheel of the projector was removed. There was a neutral density filter (NE2R10 A, Thorlabs, Inc., Newton, NJ, USA) and a polarizer (OCZ203, Beijing Optical Century Instrument Co., Ltd., Beijing, China) in front of the projector. The neutral density filter can uniformly attenuate the light, and the polarizer in front of the projector can be used in conjunction with the polarizer in front of the camera lens to eliminate specular reflections.

In order to obtain a good projection effect, the optical axis of the projector and the camera should be on the same plane and at a small angle to obtain sinusoidal structured light and reduce specular reflection. Both the camera and the projector were installed above the sample stage.

The sample stage consisted of a horizontal translation stage (MTS123, Beijing Optical Century Instrument Co., Ltd., Beijing, China) and a vertical translation stage (MVS101, Beijing Optical Century Instrument Co., Ltd., Beijing, China). Under the control of the motor controller, the sample could be adjusted to the proper position. The horizontal translation stage had a travel of 300 mm and a displacement resolution of 0.0032 mm. The vertical translation stage had a travel of 55 mm and a displacement resolution of 0.1 mm.

#### 2.1.2. Software

**Figure 2:**
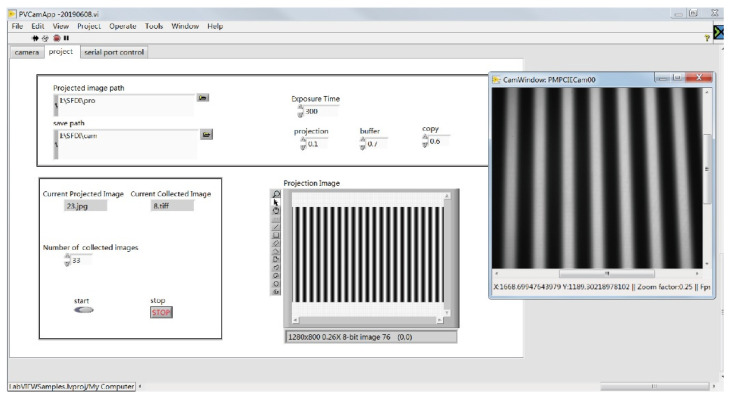
Software for data acquisition and system control developed on the LabVIEW platform.

As shown in Figure 2, the acquisition and control software of the SFDI system was developed on the LabVIEW 2018 environment (National Instruments, Austin, TX, USA). In the LabVIEW programming environment, software development kits (SDKs) provided by camera manufacturers were used to implement camera control, projection–camera acquisition synchronous operation, stage movement and camera–stage distance measurement. A histogram of a single shot image was displayed during image acquisition and could be used to determine whether the image was overexposed. The preprocessed projection image was read from the specified folder in the computer by the software written in the lab. Projection and shooting were performed sequentially, and a proper amount of delay (1.5 s) was added to prevent the order of projection and shooting from being disordered.

#### 2.1.3. System Operation

Before collecting images, the parameters of the projector should be set—sharpness, contrast, brightness, etc.—so that the projection contrast is high enough but not saturated. The projection image was pre-processed by keystone correction. The picture size was set to the projector’s standard resolution of 1280 × 800 pixels, which allowed the image to be projected without loss of resolution.

Before shooting, the path of the projection image and the path to save the image were set. The software automatically read the serial number of the picture, and read the image to project or save the captured image in order. To ensure imaging quality, the exposure time had to be set after each sample placement. A single image was taken, and the sample position was observed and adjusted to the middle of the field of view. A histogram was used to set the exposure time to increase the signal to noise ratio (SNR).

### 2.2. System Calibrations

The system calibration aimed to obtain an accurate projection of the gray response, which is calibrated by a standard whiteboard:

(1) $$R_{\mathrm{ac}}\left({x,f}_{x}\right)=\frac{I_{\mathrm{ac}}\left({x,f}_{x}\right)}{I_{\mathrm{ref}}}R_{d.\mathrm{ref}}{(f}_{x})$$

In the optical calibration and correction experiments, I_ac_(x,f_x_) is the reflected grayscale of the different frequency sinusoidal patterns projected, I_ref_ is the reflected grayscale of a full white projected pattern, R_d,ref_(f_x_) is the reflectance of a standard whiteboard (0.99 in this study) (150 × 150 mm^2^, Guangzhou Jingyi Photoelectric Technology Co., Ltd., Guangzhou, China) and R_ac_(x,f_x_) is the reflectance after calibration.

#### 2.2.1. Projector–Camera Calibration

The camera calibration was performed by MATLAB (The MathWorks, Inc., Natick, MA, USA). The external parameter matrix of the camera was used to determine if the camera was installed correctly.

After the camera calibration, a ceramic checkerboard was used for projector–camera calibration. The gray code pattern was projected onto the ceramic checkerboard and decoded to obtain the relationship between the calibration board’s corner points and the projection’s coordinates [26]. The gray code pattern was generated by Visual Studio 2017 (Microsoft Corporation, Redmond, WA, USA). After calibration, the internal parameter matrix of the projector and the camera, the external parameter matrix of the projector–camera setup, and the coordinates of the corner points on the camera image and the projected pattern could be obtained. The calibration result was judged by the reprojection error.

The relative position of the projector and the camera was determined by the external parameter matrix of the projector–camera setup, and the optical axis of the projector and the camera were adjusted to the same plane. The height *h* of the projector from the checkerboard plane and the projection angle (the pitch angle *α* and the yaw angle *β*) were given by the external parameter matrix of the camera and the projector–camera setup. The average pixel length of the checkerboard X_c,mean_ was obtained by calculating the average distance between the coordinates of all corner points.

#### 2.2.2. Keystone Correction

The existence of the projection angle will cause keystone distortion of the projected pattern. The projection angles are the pitch angle *α*, the yaw angle *β* and the roll angle. In actual installation, the pitch angle is inherent to the system, but the yaw angle and the roll angle should be avoided as much as possible. The hardware design of this study can avoid the existence of the roll angle. Therefore, in this study, a small amount of yaw error was considered, and the roll angle error was ignored to simplify the calculation formula. The effect of the pitch angle *α* and the yaw angle *β* on the projection was mainly considered, and the effect of keystone distortion was minimized through error parameters.

The method of projector keystone correction in this study is similar to the inverse perspective transform [27]. The original projection image was used to generate a corrected projection image through inverse perspective transformation, and then an image without keystone distortion was projected. The specific steps were as follows:

① Use the projector’s calibration parameters and the original image to generate point coordinates of the world coordinates plane, where the set angle was opposite to the actual angle of the projector.
② Convert the scattered point coordinates into pixel coordinates to obtain a corrected projection image.
③ Calculate the error and set the projection image at different angles until the error has been minimized.

A simplified correction formula can be obtained when the roll angle is ignored. P_i_ = {p_p_, q_p_, 1, 1} is a point in the image plane of projector, p_p_ and q_p_ represent pixel coordinates, and P^g^ is a point on the corresponding checkerboard plane. The relationship between the two is P^g^ = $T_{i}^{g}$P^i^, where:

(2) $$T_{i}^{g}=h\left[\begin{array}{cccc} -\frac{1}{f_{u}}c_{2} & \frac{1}{f_{v}}s_{1}s_{2} & \frac{1}{f_{u}}c_{u}c_{2}-\frac{1}{f_{v}}c_{v}s_{1}s_{2}-c_{1}s_{2} & 0\\ \frac{1}{f_{u}}s_{2} & \frac{1}{f_{v}}s_{1}c_{1} & -\frac{1}{f_{u}}c_{u}s_{2}-\frac{1}{f_{v}}c_{v}s_{1}c_{2}-c_{1}c_{2} & 0\\ 0 & \frac{1}{f_{v}}c_{1} & -\frac{1}{f_{v}}c_{v}c_{1}+s_{1} & 0\\ 0 & -\frac{1}{hf_{v}}c_{1} & \frac{1}{hf_{v}}c_{v}c_{1}-\frac{1}{h}s_{1} & 0 \end{array}\right]$$

where *α* is the pitch angle; *β* is the yaw angle; c_1_ = cos*α*; s_1_ = sin*α*; c_2_ = cos*β*; s_2_ = sin*β*; *h* is the height of the projector from the checkerboard plane, given by the external parameter matrix; and f_u_, f_v_, c_u_ and c_v_ represent the horizontal focal length, the vertical focal length, horizontal optical center coordinates and the vertical optical center coordinates of the projector, respectively, given by the internal parameter matrix of the projector.

P^g^ = {x_g_, y_g_, −*h*, 1} represents the world coordinate point, which is also a point on the checkerboard plane. The corrected image coordinate point Pic is calculated by P^ic^ = $T_{g}^{i}$P^g^, where:

(3) $$T_{g}^{i}=\left[\begin{array}{cccc} f_{u}c_{2}{+c}_{u}c_{1}s_{2} & c_{u}c_{1}c_{2}-s_{2}f_{u} & -c_{u}s_{1} & 0\\ s_{2}{(c}_{v}c_{1}-f_{v}s_{1}) & c_{2}{(c}_{v}c_{1}-f_{v}s_{1}) & -f_{v}c_{1}-c_{v}s_{1} & 0\\ c_{1}s_{2} & c_{1}c_{2} & {-s}_{1} & 0\\ c_{1}s_{2} & c_{1}c_{2} & {-s}_{1} & 0 \end{array}\right]$$

Through this inverse process, pixel coordinates can be obtained from world coordinates. After two steps of transformation, the original image can be transformed into a keystone correction pattern.

The error is the average of the standard deviation of pixels in the vertical fringe direction, that is, for an image with a m × n pixel area with a horizontal fringe:

(4) $$e_{p}=\sum_{i=1}^{n}\frac{{\mathrm{std}}_{\mathrm{cow}}\left(i\right)}{n}$$

where std_cow_(i) represents the normalized pixel standard deviation of the i-th column.

Different pitch angles *α* and yaw angles *β* around the calibrated angle were set to generate the corresponding keystone corrected patterns. After obtaining the reflectance image, we calculated the keystone correction error by Equations (3) and (4) to find the optimal keystone correction angle.

#### 2.2.3. Frequency Calibration

Unlike the traditional 3D reconstruction of structured light, SFDI projects sinusoidal structured light onto the surface of the object. It studies the process of projecting a sinusoidal surface light source to a highly scattering object. The calculations of *μ_a_* and *μ’_s_* are frequency sensitive [28], so the frequency at which the sinusoidal patterns are actually generated needs to be calibrated.

For frequency calibration, an image of a ceramic checkerboard placed horizontally on the sample stage and perpendicular to the camera’s optical axis was required. When whiteboard or liquid phantom images are collected, the height of the vertical translation stage needs to be adjusted so that the collection plane is at the same height to ensure that the actual frequency does not change.

The camera calibration results can be used to calculate the average pixel length X_c,mean_ of the checkerboard. The actual period of the projected pattern can then be calculated by using Equation (5):

(5) $$\frac{T_{r}}{T_{c}}=\frac{X_{r}}{X_{c,\mathrm{mean}}}$$

where T_r_ represents the actual period of the projected pattern, T_c_ represents the pixel period of the image captured by the camera, X_r_ represents the actual cell length of the checkerboard (5 mm in this study) and X_c,mean_ represents the average pixel length of the checkerboard image.

The ratio of the fringe generation period T_p_ to the actual period T_r_ is:

(6) $$r_{r}^{p}=\frac{T_{p}}{T_{r}}$$

When the actual period required is known, the pixel period can be obtained by the period ratio. First, a sinusoidal pattern was preset according to the actual projection situation (the size of the pattern actually projected on the sample) with a period of T_p_(0) = 40 pixels. This was projected onto a standard whiteboard to calibrate the image reflectance. A region of interest (ROI) was selected and averaged along the direction of the vertical fringes to obtain a sinusoidal average reflectance curve. The number of fringe periods of the ROI was calculated by the number of minimum points on the curve. T_c_ was calculated by the pixel coordinates of the minimum points. The period ratio $r_{r}^{p}$(0) could then be calculated.

Sixteen reference frequencies were used: 0 mm^−1^, 0.007 mm^−1^, 0.0084 mm^−1^, 0.0098 mm^−1^, 0.0112 mm^−1^, 0.0126 mm^−1^, 0.014 mm^−1^, 0.028 mm^−1^, 0.042 mm^−1^, 0.056 mm^−1^, 0.070 mm^−1^, 0.084 mm^−1^, 0.098 mm^−1^, 0.112 mm^−1^, 0.126 mm^−1^ and 0.14 mm^−1^. The expected actual period is the reciprocal of the reference frequency. By using $r_{r}^{p}$(0) and the 16 expected actual periods T_re_(k) (k = 1,2…,16), the 16 projection periods T_p_(k) were calculated by Equation (6).

In the first step, the 16 generated images were projected and T_c_(k) was calculated. The 16 actual period verification values T_v_(k) were then calculated by Equation (5). Corresponding to the expected actual period T_re_, the period percentage error e_t_ was:

(7) $$e_{t}=\left|\frac{T_{\mathrm{re}}-T_{v}}{T_{\mathrm{re}}}\right|\times 100\%$$

In the second step, we substituted T_v_ as T_r_ into Equation (6) and updated the 16 corresponding period ratios $r_{r}^{p}$(k). Using the updated period ratios $r_{r}^{p}$(k) and the expected actual period T_re_(k), the projection period T_p_(k) was calculated again. The actual period verification values T_v_(k) and the relative period error e_t_(k) were then calculated again.

#### 2.2.4. Calibration of the Optical Properties 

Phantoms are often used to verify the accuracy of a system or an algorithm. In this study, TiO_2_ (T104950-500 g, Aladdin, Shanghai, China) was used as the scattering agent, and Indian ink (Royal Talens, Apeldoorn, The Netherlands) was used as the absorber. Different concentration gradients were set to linearly correct *μ_a_* and *μ’_s_*.

In total, 6 wavelengths were used in this study: 460 nm, 503 nm, 527 nm, 630 nm, 658 nm and 675 nm. Two sets of liquid phantoms were set up, and each set of liquid phantoms had 9 samples. Liquid phantoms in the first set were labeled #1 to #9, the TiO_2_ volume fraction was 0.1%, and the ink volume fraction ranged from 0.004% to 0.02% (with an interval of 0.002%). Liquid phantoms in the second set were labeled #10 to #18, the ink volume fraction was 0.006% and the TiO_2_ volume fraction ranged from 0.04% to 0.2% (with an interval of 0.02%).

The reference value of *μ_a_* of the liquid phantoms were calculated by the Lambert–Beer law. A spectrometer (QE65pro, Ocean Insight Co., Ltd., Orlando, FL, USA) was used to measure the collimated transmittance T of the pure absorption sample, and *μ_a_* was calculated by:

(8) $$\mu_{a}=\frac{-\ln(T)}{d}=\frac{-\ln(I/I_{0})}{d}$$

where d is the optical path length of the pure absorption solution.

The reduced scattering coefficient *μ’_s_* of the liquid phantoms was calculated by Mie scattering simulation. According to the refractive index, the volume fraction, the diameter of the TiO_2_, the refractive index of deionized water and the wavelength, reference *μ’_s_* values can be obtained by using the Mie program [29] based on MATLAB.

The three-phase pattern with a projection frequency of f_x_ was applied to the liquid phantoms. The three-phase demodulation was performed by using Equation (9), and the reflectance was calibrated by the standard whiteboard using Equation (10):

(9) $$M_{\mathrm{ac}}\left({x,f}_{x}\right)=\frac{\sqrt{2}}{3}\sqrt{{{(I}_{1}-I_{2})}^{2}{+(I}_{2}-I_{3}{)}^{2}{+(I}_{1}-I_{3}{)}^{2}}$$

(10) $$R_{d}\left({x,f}_{x}\right)=\frac{M_{\mathrm{ac}}\left({x,f}_{x}\right)}{M_{\mathrm{ac},\mathrm{ref}}\left({x,f}_{x}\right)}R_{d.\mathrm{ref}}{(f}_{x})$$

where I_1_, I_2_ and I_3_ represent the reflection intensity of the sample collected by the three-phase projected pattern, R_d_(x, f_x_) represents the reflectance after calibration, R_d,ref_(f_x_) represents the reflectance of the standard whiteboard, M_ac_(x, f_x_) represents the amplitude envelope of the diffuse reflection light collected by the sample and M_ac,ref_(x, f_x_) represents the amplitude envelope of the diffuse reflection light collected by the standard whiteboard.

After the reflectance had been calibrated, multi-frequency fitting inversion was performed by Equation (11).

(11) $$R_{d}\left(f_{x}\right)=\frac{{3A\mu }^{’}{}_{s}/\mu_{tr}}{\left(\mu^{’}{}_{eff}/\mu_{tr}+1\right)\left(\mu^{’}{}_{eff}/\mu_{tr}+3A\right)}$$

where:

(12) $$A=\frac{{1-R}_{\mathrm{eff}}}{2\left({1+R}_{\mathrm{eff}}\right)}$$

(13) $$R_{\mathrm{eff}}=0.636n+0.668+0.71/n-1.44{/n}^{2}$$

(14) $$\mu^{’}{}_{eff}=\sqrt{\mu_{eff}{}^{2}{+(2\pi f}_{x}{)}^{2}}$$

(15) $$\mu_{eff}=\sqrt{{3\mu }_{a}{(\mu }_{a}{+\mu }_{s}^{’})}$$

where R_d_(f_x_) represents the reflectance after calibration, A represents the proportional constant, R_eff_ represents the effective reflection coefficient, n represents the refractive index. *μ’_eff_* represents the scalar attenuation coefficient in the spatial frequency domain, *μ_eff_* represents the effective attenuation coefficient and *μ_tr_* = *μ_a_* + *μ’_s_* represents the full attenuation coefficient, where *μ_a_* and *μ’_s_* refer to the absorption coefficient and the reduced scattering coefficient, respectively.

For a set m of phantoms, the linearity error of the system due to TiO_2_ precipitation and other reasons was corrected by:

(16) $$k{\left(\mu_{a}\right)}_{\lambda }=\sum_{i=1}^{m}\frac{\mu_{a}{\left(i\right)}_{\mathrm{ref}}{/\mu }_{a}{\left(i\right)}_{\mathrm{mea}}}{m}$$

(17) $${k(\mu }_{s}^{’}{)}_{\lambda }=\sum_{j=1}^{m}\frac{\mu_{s}^{’}{\left(j\right)}_{\mathrm{ref}}{/\mu }_{s}^{’}{\left(j\right)}_{\mathrm{mea}}}{m}$$

where *μ_a_*(*i*)_ref_ and *μ_a_*(*i*)_mea_, respectively, represent the reference values and measured values of *μ_a_* for the i-th phantom; *μ’_s_*(*j*)*_ref_* and *μ’_s_*(*j*)*_mea_* represent the reference values and measured values of *μ’_s_* for the j-th phantom, respectively; and k(*μ_a_*)_λ_ and k(*μ’_s_*)_λ_ represent the correction ratio of *μ_a_* and *μ’_s_*, respectively.

Therefore:

(18) $$\mu_{a,cal}{=k(\mu }_{a}{)}_{\lambda }\mu_{a,mea}$$

(19) $$\mu_{s,cal}^{’}{=k(\mu }_{s}^{’}{)}_{\lambda }\mu_{s,mea}^{’}$$

where, respectively, *μ_a__,cal_* and *μ’_s__,mea_* represent the calibrated values and measured values of *μ_a_* for the phantoms, and *μ’_s__,cal_* and *μ’_s__,mea_* represent the calibrated values and measured values of *μ’_s_* the phantoms.

### 2.3. Sample Preparation

**Figure 3:**
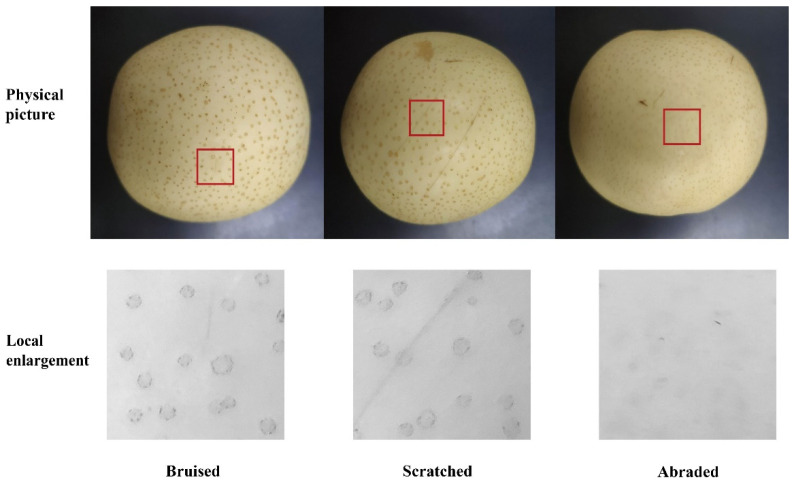
Bruised, scratched and abraded pears and the corresponding local enlargements.

Eighty normal crown pears with no surface defects were purchased from a local fruit store and placed in a laboratory at 19 °C (room temperature) and 55% humidity for about 24 h. All pears were equally divided into four groups: Group A was the normal group, without any treatment. Group B was the bruise group. In a pendulum device, a 20 mm diameter steel ball with a weight of 31.3995 g was released from a height of 0.3 m and hit the equatorial part of the pear to cause slight bruises. Group C was the scratch group. A razor blade was used to make a few light strokes near the equator of the pear to produce shallow scratches. Group D was the abrasion group, for which sandpaper was gently rubbed a few times near the equator of the pear, causing a slight abrasion (Figure 3).

### 2.4. Discriminant Model Analysis

Linear discriminant analysis (LDA) is a supervised pattern recognition method [30], which mainly projects high-dimensional pattern samples into the optimal discriminative vector space to extract classification information and compress the dimensionality of the feature space. Optimization analysis was performed using the Statistics and Machine Learning Toolbox 11.3 in MATLAB 2018a.

Two LDA models were compared to assess the accuracy of the classification of pears with different impairments using SFDI with spatially modulated light. The first model used data from a subset of the SFDI data. The images were acquired when the spatial frequency was 0 mm^−1^, which represents the dataset under conventional planar light irradiation. The second model used the full SFDI dataset.

A ROI of 400 × 400 pixels (26 × 26 mm^2^) was selected for data processing, and a region of 200 × 200 pixel points was generated by 2 × 2 binning to reduce the time for data processing. Each sample was labeled and trained in the four categories (A (normal pear), B (bruised pear), C (scratched pear) and D (scuffed pear)) as described above after inversion to obtain *μ_a_* and *μ’_s_*, and then averaged. The accuracy of the model was determined using k-fold cross-validation for 5 folds.

## 3. Results and Discussion

### 3.1. Projector–Camera Calibration Results

Via Equation (1), the projected normalized reflectance image was obtained by a standard whiteboard. Images of a standard whiteboard were obtained under a white field and from projections at set frequencies.

**Figure 4:**
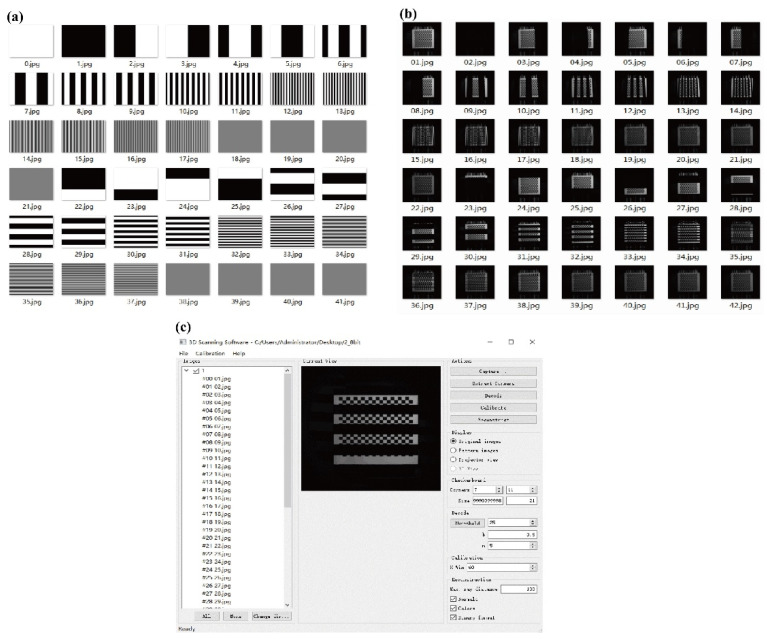
Gray code patterns: (**a**) generated gray code pattern; (**b**) captured image; (**c**) projector calibration software.

As shown in Figure 4a, a total of 21 gray code patterns and their complementary images were generated. The generated gray code pattern was projected onto a ceramic checkerboard calibration board, as shown in Figure 4b. By changing the angle of the calibration plate, and shooting about 10 groups in total, the result was considered to be more accurate. Gray code decoding and projector calibration were realized through open-source software [26]. The calibration software interface is shown in Figure 4c. The reprojection error of the projector was 0.63 pixels, and the reprojection error of the camera was 2.32 pixels. The reprojection error of the camera here was larger than the results obtained by Moreno and Taubin [26]; this may be due to the fact that the parameters in the program cannot be configured when using the software, but the reprojection error of the projector was similar. Overall, the projector–camera calibration results are reliable.

After calibration, it was found that X_c,mean_ = 78.3 pixels, *h* = 527 mm, *α* = 18°, *β* = 0.6° and the internal parameter matrix of the projector was (2116.8, 0, 670.4; 0, 2122.4, −186.8; 0, 0, 1).

### 3.2. Keystone Correction Results

**Figure 5:**
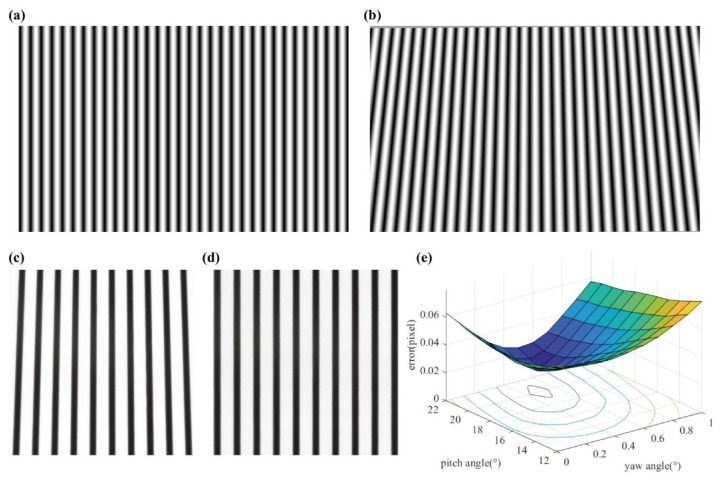
Keystone correction: (**a**) sinusoidal pattern before correction; (**b**) sinusoidal pattern after correction; (**c**) ROI reflectance image before correction; (**d**) ROI reflectance image after correction; (**e**) correction error gradient map.

Figure 5a shows the sinusoidal pattern without keystone correction, and Figure 5b shows the keystone-corrected pattern. The keystone-corrected pattern was projected onto a standard whiteboard. The reflectivity of the image was calculated by Equation (1), and the reflectivity of the image was used to determine the correction effect. Figure 5c shows the reflectivity of the image before correction, and Figure 5d shows the reflectivity of the image after correction. The range of *α* was 12°–22°, with gradient of 1°. The range of *β* was 0°–1°, with a gradient of 0.1°. As shown in Figure 5e, the minimum correction error e_p_ = 0.017 pixels, the corresponding pitch angle *α* = 20° and the yaw angle *β* = 0.5°. If the angle gradient were smaller, the number of images to be acquired would grow. If the roll angle were added, the equation would become very complicated. However, the hardware design of this study can also avoid the existence of a roll angle. The error after keystone correction was very small, and it can be considered that the captured image has no obvious deformation. After simplifying the correction process, the keystone can still be corrected accurately.

### 3.3. Frequency Calibration Results

After keystone correction, the actual frequency of the sinusoidal pattern was calibrated. The average pixel period of the captured image T_c_(0) was 158.2 pixels. The calculation showed that the actual period of the projected pattern T_r_(0) = 10.1 mm, and the period ratio $r_{r}^{p}$(0) = 3960 pixel m^−1^. The calculated value $r_{r}^{p}$(0) was used as the initial period ratio, and the required projection pixel period was calculated. The verification results were obtained at each frequency, and the period ratio $r_{r}^{p}$(k) was updated at each frequency by Equations (5) and (6) and re-checked after the projection collection.

**Figure 6:**
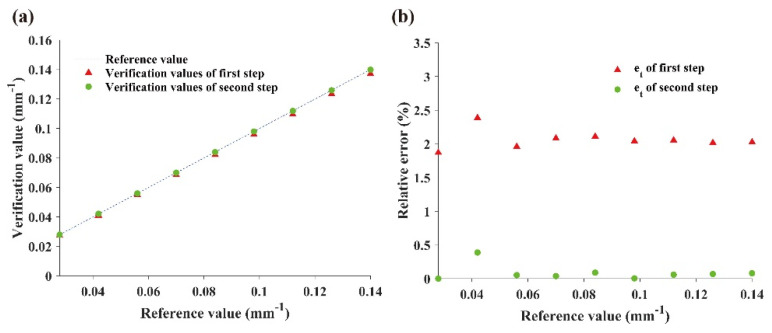
Calibration of projection frequency: (**a**) expected frequency and the two-step verification values; (**b**) percentage errors of two-step validation.

When the frequency was less than 0.014 mm^−1^, a complete sinusoidal pattern could not be captured on the whiteboard. Therefore, only nine frequencies larger than 0.014 mm^−1^ were used for the calibration; other frequencies were estimated according to the frequency calibration results. Frequency is the reciprocal of the period. The relationship between the expected frequency and its two-step verification values at each frequency is shown in Figure 6a. The relative error of period at each frequency was calculated by Equation (7). As shown in Figure 6b, the average percentage error after calibration reduced from 2.1% to 0.1%.

### 3.4. Optical Property Calibration Results

**Figure 7:**
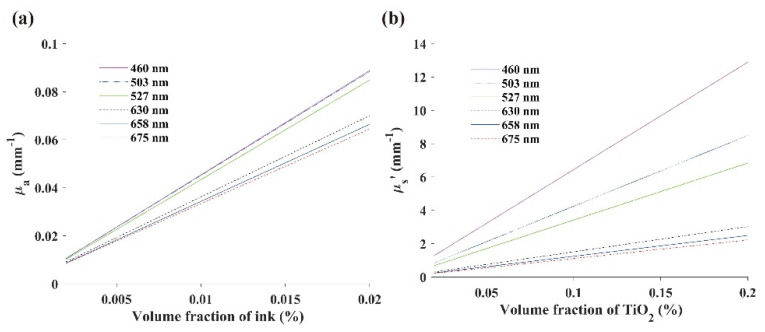
Reference values for phantom optical properties: (**a**) reference values of *μ_a_*; (**b**) reference values of *μ’_s_*.

Liquid phantoms were used to calibrate the optical properties measured by the SFDI system. The reference values of *μ_a_* and *μ’_s_* of the phantoms were calculated at six wavelengths. The relationship between the reference value of the optical properties and the volume fraction is shown in Figure 7. The reference value of *μ_a_* was calibrated linearly. The reference values of *μ’_s_* was related linearly to the volume fraction of TiO_2_. Both *μ_a_* and *μ’_s_* increased with increasing wavelengths.

The determination coefficients of the measured absorption coefficient *μ_a_* and the ink volume fraction at six wavelengths are shown in Table 1. This shows that the reference values of *μ_a_* and the volume fraction of ink are highly linearly related.

The projection image was corrected and calibrated. The captured images were demodulated in three phases after the ROI (300 × 300 pixels) was selected, and the reflectance was calibrated. Multi-frequency fitting was used for inversion. After the optical properties had been obtained, the measured values and the reference values were fitted linearly to eliminate the linearity error of the system: the first set of data was used for *μ_a_* calibration and the *μ’_s_* test; the second set of data was used for the *μ_a_* test and *μ’_s_* calibration.

The average error of the six selected wavelengths is shown in Table 2. The overall average error of *μ_a_* and *μ’_s_* at six wavelengths was less than 8.88% and 4.54%, respectively, which is slightly better than the values reported by Matthew et al. in their recent study (23% and 6%) [31]. Linear calibration of the liquid phantoms can make the measured value of the sample closer to the true value. There is still some error in *μ_a_* and *μ’_s_* after the linear calibration, which means that there are still other non-linear errors, such as an unstable light source [32], and theoretical errors of diffusion approximation [33], which have noticeable impacts on the measurement of *μ_a_*. In this study, the inversion method of multi-frequency fitting was adopted. Other improved inversion methods [17] can be adopted in further research.

### 3.5. Damage Discrimination Results

**Figure 8:**
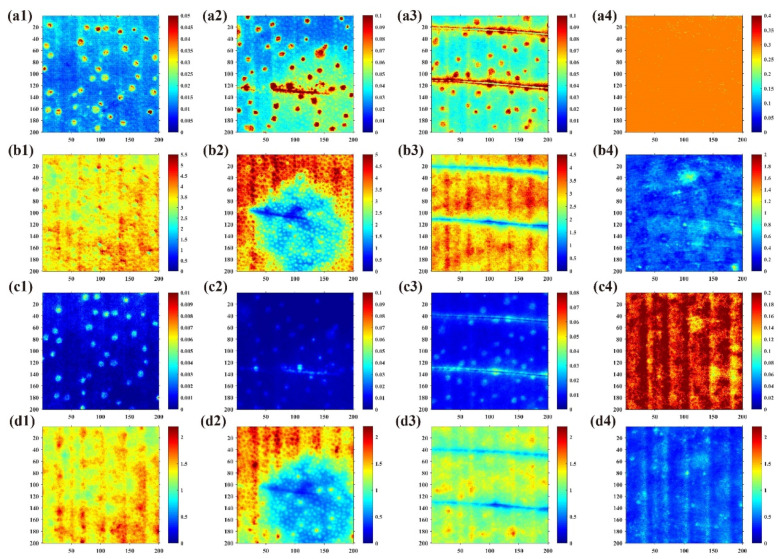
(**a1**–**a4**) and (**b1**–**b4**) are the *μ_a_* and *μ’_s_* maps of normal, bruised, scratched, and abraded pears at 527 nm, respectively; (**c1**–**c4**) and (**d1**–**d4**) are the corresponding *μ_a_* and *μ’_s_* maps at 675 nm, respectively.

Figure 8 shows the *μ_a_* and *μ’_s_* pseudo-color plots of normal pears and three different damage types (bruised, scratched and abraded) at 527 and 675 nm. It can be seen from the figure that the *μ_a_* of the damaged region is larger than that of the normal area, while *μ’_s_* is the opposite, which is similar to other results in the literature [6,17]. Compared with *μ_a_* mapping, the type of damage is easier to identify in *μ’_s_* mapping and can be roughly distinguished. This is due to the fact that a pear generally undergoes physical structural changes when damage occurs, which affects the *μ’_s_* of the sample [18]. It can also be seen that at 527 nm, both *μ_a_* and *μ’_s_* are larger than at 675 nm, which is due to the fact that the pear has a smaller absorption peak near 527 nm, while the *μ’_s_* of the pear tapers off in visible (VIS) and near-infrared (NIR) range [34]. Secondly, it can be seen that there are many spots in the figure, which are the pear’s epidermal breathing pores.

From the results (Table 3) of the four classifications (normal, bruised, scratched and abraded), it can be seen that the SFDI technique was better at detecting and classifying pears with different damage types compared with the traditional planar light technique. The classification accuracy at 527 nm was higher than that at 675 nm, which was probably the result of the absorption peak of pears near 527 nm. When the classification results were analyzed, it was found that bruised and scratched pears were misclassified more often, which may be because both belong to minor damage and thus the average *u_a_* and *u’_s_* values in the region are close. Hence, the pears were classified into three classes: normal, minor damage and serious damage, and the same LDA method was used to classify them. The results showed that the classification of the new method was more accurate; in particular, the classification accuracy of the SFDI detection technique at 527 nm was up to 100%. In the study of Zhang et al. [6], the optical properties of apples with different levels of bruising were measured using integrating spheres and classified based on this, with an accuracy of 92.5%. This further illustrates the feasibility of using optical properties for non-destructive detection of early fruit damage.

Compared with the reference literature [18,35], the *μ_a_* obtained in this experiment is small and the *μ’_s_* is large. In order to further explore the reason for this, the peel of the same batch of samples was removed for flesh image processing, and it was found that the peel has a certain influence on the *μ_a_* and *μ’_s_* of pears, which was reported in the study of Hu et al. [36]. In addition, the experiment was only carried out at two wavelengths (527 and 675 nm) and the near infrared part was not studied due to system limitations; however, the pear had significant absorption peaks in the near infrared part [34], and the SFDI system could be improved to carry out more wavelength experiments in the future.

## 4. Conclusions

In this study, an optical calibration and correction method was proposed for the SFDI system. After optical calibration and correction, the accuracy of the system’s measurement was verified by using liquid phantoms. Projector–camera calibration, projection keystone correction and frequency calibration ensured that the projected pattern was not distorted and made the experimental conditions closer to the ideal experimental conditions to reduce the experimental error. Next, the optical parameters of normal pears and three different damage types of pears were measured using the calibrated SFDI system, and the LDA method was used for discrimination of pears with different surface damage types based on the obtained *μ_a_* and *μ’_s_*. Further studies can be implemented for investigating the application prospects of the SFDI technique for the detection of agricultural products and foodstuffs.

## Figures and Tables

**Table 1:** Determination coefficients of the measured *μ_a_* and the ink volume fraction at six wavelengths.

Wavelength (nm)	460	503	527	630	658	675
R^2^	0.9955	0.9964	0.9961	0.9965	0.9965	0.9963

**Table 2:** Average error of the measured optical properties at six wavelengths.

Wavelength (nm)		460	503	527	630	658	675	Average
**Relative error (%)**	*μ_a_*	6.92	8.47	8.54	11.03	10.12	8.23	8.88
	*μ’_s_*	4.02	5.08	4.55	6.21	4.76	2.6	4.54

**Table 3:** The five-fold cross-validation outcomes for two models.

Wavelength (nm)			527	675
Cross-validation accuracy for the training set (%)	Four categories (bruised, scratched and abraded)	0 mm^−1^ (planar)	82.5	77.5
		All spatial frequencies (SFDI)	92.5	83.8
	Three categories (normal, minor damage and serious damage)	0 mm^−1^ (planar)	93.8	93.8
		All spatial frequencies (SFDI)	100	98.8

## Data Availability

The data that support the findings of this study are available from the corresponding author upon reasonable request.
